# *Leishmania donovani* Nucleoside Hydrolase Terminal Domains in Cross-Protective Immunotherapy Against *Leishmania amazonensis* Murine Infection

**DOI:** 10.3389/fimmu.2014.00273

**Published:** 2014-06-11

**Authors:** Dirlei Nico, Daniele Crespo Gomes, Iam Palatnik-de-Sousa, Alexandre Morrot, Marcos Palatnik, Clarisa Beatriz Palatnik-de-Sousa

**Affiliations:** ^1^Laboratório de Biologia e Bioquímica de Leishmania, Departamento de Microbiologia Geral, Instituto de Microbiologia Paulo de Góes, Universidade Federal do Rio de Janeiro, Rio de Janeiro, Brazil; ^2^Programa de Pós Graduação em Metrologia, Laboratório de Biometrologia, Pontifícia Universidade Católica do Rio de Janeiro, Rio de Janeiro, Brazil; ^3^Laboratório de Imunologia, Instituto de Microbiologia Paulo de Góes, Universidade Federal do Rio de Janeiro, Rio de Janeiro, Brazil; ^4^Programa de Pós Graduação em Clínica Médica, Faculdade de Medicina, Universidade Federal do Rio de Janeiro, Rio de Janeiro, Brazil

**Keywords:** visceral leishmaniasis, cutaneous leishmaniasis, diffuse cutaneous leishmaniasis, cross- immunotherapy, nucleoside hydrolases, recombinant vaccines

## Abstract

Nucleoside hydrolases of the *Leishmania* genus are vital enzymes for the replication of the DNA and conserved phylogenetic markers of the parasites. *Leishmania donovani* nucleoside hydrolase (NH36) induced a main CD4^+^ T cell driven protective response against *L. chagasi* infection in mice which is directed against its C-terminal domain. In this study, we used the three recombinant domains of NH36: N-terminal domain (F1, amino acids 1–103), central domain (F2 aminoacids 104–198), and C-terminal domain (F3 amino acids 199–314) in combination with saponin and assayed their immunotherapeutic effect on Balb/c mice previously infected with *L. amazonensis*. We identified that the F1 and F3 peptides determined strong cross-immunotherapeutic effects, reducing the size of footpad lesions to 48 and 64%, and the parasite load in footpads to 82.6 and 81%, respectively. The F3 peptide induced the strongest anti-NH36 antibody response and intradermal response (IDR) against *L. amazonenis* and a high secretion of IFN-γ and TNF-α with reduced levels of IL-10. The F1 vaccine, induced similar increases of IgG2b antibodies and IFN-γ and TNF-α levels, but no IDR and no reduction of IL-10. The multiparameter flow cytometry analysis was used to assess the immune response after immunotherapy and disclosed that the degree of the immunotherapeutic effect is predicted by the frequencies of the CD4^+^ and CD8^+^ T cells producing IL-2 or TNF-α or both. Total frequencies and frequencies of double-cytokine CD4 T cell producers were enhanced by F1 and F3 vaccines. Collectively, our multifunctional analysis disclosed that immunotherapeutic protection improved as the CD4 responses progressed from 1+ to 2+, in the case of the F1 and F3 vaccines, and as the CD8 responses changed qualitatively from 1+ to 3+, mainly in the case of the F1 vaccine, providing new correlates of immunotherapeutic protection against cutaneous leishmaniasis in mice based on T-helper TH1 and CD8^+^ mediated immune responses.

## Introduction

Leishmaniasis is a complex of vector-borne protozoan diseases the etiological agents of which belong to the *Leishmania* genus. The global incidence and prevalence of leishmaniasis is increasing. The main clinical syndromes of leishmaniasis are: cutaneous (CL), diffuse cutaneous (DCL), mucocutaneous (MCL), and visceral (VL) (1). While CL accounts for approximately 0.7–1.2 million cases per year, which is more than 50% of the new cases of leishmaniasis (2). Most of the CL cases occur in the Mediterranean (85,555 cases/year), the Americas (66,941 cases/year), and the Middle East to Central Asia (61,013 cases/year) (2). The 10 countries with the highest estimated number of cases are: Afghanistan, Algeria, Colombia, Brazil, Iran, Syria, Ethiopia, North Sudan, Costa Rica, and Peru and together they account for 70–75% of the estimated global incidence of CL (2). The disease causes skin ulcers at the site of the sand-fly bite, usually on exposed parts of the body, such as the face, neck, arms, and legs and develops an active T cell mediated immune response that plays a pivotal role in the processes in the cure or in the aggravation of the disease (3). VL, on the other hand, has approximately 0.2–0.4 million new cases per year (2) and is the most severe clinical syndrome of leishmaniasis characterized by hepato-splenomegaly, malaise, cachexia, fever, hypergammaglobulinemia, anemia, and the progressive suppression of the T cell mediated immune response. If left untreated, the disease has a high mortality rate mainly due to immunosuppression and secondary infections. Indeed, anergy to leishmanial antigens and negative skin tests have been reported in cases of VL caused by *Leishmania donovani* and *L. infantum/chagasi* (4–6), and DCL caused by *L. amazonensis* (7) while a strong TH1 pro-inflammatory response has been detected in cases of CL (8) and MCL caused by *L. braziliensis* (9).

Since the chemotherapy of leishmaniasis is highly toxic and the few available therapeutic drugs are only partially effective (10, 11), due to an increase in the resistance of parasites to antibiotics, a protective vaccine would be important not only for prophylaxis but also for the immunotherapy of the disease. The success of immunotherapy in the control of human CL leishmaniasis with the use of crude parasite vaccines combined to BCG has been reported since the 80s (12–14). Furthermore, immunochemotherapy against human CL leishmaniasis has been reported to reduce the time of chemotherapy needed to cure this disease in humans, thus decreasing its toxicity (15).

Since the epidemics of VL and CL are spreading on a worldwide scale, even overlapping in some areas, and no human vaccine is available yet, the development of a bivalent vaccine for the control of tegumentary and VL leishmaniasis is highly recommended. Consequently, we believe that the search for cross-protective antigens is mandatory. Recently, we developed the first licensed second generation vaccine against canine VL leishmaniasis (Leishmune^®^), which contains the fucose–mannose ligand (FML) antigen of *L. donovani* in formulation with saponin (16–19), is a transmission blocking vaccine (18, 19) and has already determined a reduction in the incidence of the human and canine disease in Brazilian endemic areas (20). Prophylactic vaccination of dogs with Leishmune^®^ promoted increases in the production of NO, IgG2 antibodies against FML and *L. chagasi*, intradermal reactions and proportions of CD8^+^ lymphocytes, which secrete more IFN-γ than IL-4 (21, 22) expressing a selective pro-inflammatory pattern (IFN-γ/NO) (23). The early and persistent activation of neutrophils and monocytes have also been described (23). This increase in proportions of CD8^+^ T cells is expected for the QS21 saponin adjuvant of Leishmune^®^ (24) and this was also described in the Leishmune^®^ immunotherapy assays against naturally (25) and experimentally acquired canine VL leishmaniasis (26). Furthermore, the sustained or increased proportions of CD4^+^ and CD21-B lymphocytes (25, 26) and the reduced CD4^+^/CD25^+^ T cell counts (27) have also been described in Leishmune^®^ vaccinated dogs.

Leishmune^®^ canine immunotherapy, on the other hand, reduced the number of deaths and the clinical and parasitological signs of canine VL and, when used for immunochemotherapy with allopurinol, amphotericin, and enrofloxacin, promoted the sterile cure (28).

QS21 and deacylated saponins of *Quillaja saponaria* are the adjuvants of the Leishmune^®^ vaccine (29). The QS21 Stimulon 1 saponin (Agenus) is also the adjuvant currently being studied in 17 human clinical programs, including four Phase 3 anti-Malaria assays, by GlaxoSmithKline. The anti-Malaria vaccine, called the RTS,S or Mosquirix, indeed contains the *P. falciparum* cir-cumsporozoite (CS) protein central tandem repeat and carboxy-terminal regions fused to the amino-terminus of the S antigen of hepatitis B virus (HBsAg) (30) and the AS01 adjuvant, which is composed of QS21 Stimulon in combination with monophosphoryl Lipid A (31). The RTS,S/AS01 vaccine co-administered with EPI vaccines provided modest protection against both clinical and severe malaria in young infants (32).

The main component of the FML antigen is the nucleoside hydrolase of *L. donovani* (NH36), which was the only FML component specifically recognized by the sera of patients with human VL leishmaniasis (33). NH36 is not only a vital enzyme which cleaves exogenous nucleosides to release pyrimidines or purines for the DNA synthesis and further replication of the parasite (34, 35), but also a strong antigen (36) present in the early stages of the parasite infection. It fulfills the requirements for a cross-protective antigen of a *Leishmania* vaccine perfectly since it is a strong phylogenetic marker *Leishmania* (37, 38) that shares high identity with the sequences of the nucleoside hydrolases of *L. major* (95%) (39), *L. mexicana* (93%), *L. chagasi* (99%), *L. infantum* (99%), *L. tropica* (97%), and *L. braziliensis* (84%) (40). This fact explains why a vaccine containing NH36, in its native form, reduced the infection by *L. donovani* (41) in mice and was characterized as an *L. major* exo-antigen (42), and in its recombinant or DNA formulations, protected mice against challenge with *L. chagasi*, *L. mexicana* (43, 44), *L. amazonensis* (45), and *L. major* (42), and dogs against challenge with *L. chagasi* (46). The DNA-NH36 vaccine induced a TH1 immune response related to the IFN-γ expression by CD4^+^ T cells, which led to an 88% prophylactic protection against VL (43), 65–81% against tegumentary leishmaniasis (42, 43, 45) and 91% immunotherapy against VL leishmaniasis in the mouse model (47). Also, higher proportions of CD4^+^-NH36-specific lymphocytes and higher levels of IFN-γ and IL-2 were found in *L. chagasi* infected dogs treated with NH36-DNA vaccine (46).

We recently obtained three recombinant fragment proteins representing the whole sequence of NH36: amino acids 1–103 (F1, N-terminal domain), 104–198 (F2, central domain), and 199–314 (F3, C-terminal domain) and used them in a mouse vaccination against *L. chagasi* infection in order to map the domain of NH36, which is the target of the adaptive immunity (48). Protection against *L. chagasi* infection in mice was determined by the C-terminal domain of NH36, which induced a main CD4^+^ T cell mediated response with a minor contribution of CD8^+^ T cells. Protection induced by this C-terminal peptide was superior to that induced by the whole protein. Vaccination with the C-terminal determined the increases of antibody titers (IgM, IgG2a, IgG1, and IgG2b), frequencies of CD4^+^ T lymphocytes, and levels of IFN-γ in the splenocyte supernatants. The proportions of CD4^+^ and CD8^+^ T lymphocytes generating IFN-γ were higher than those generating IL-10. Antibodies of Leishmune^®^ vaccinated dogs showed the most potent reactivity against the epitopes of the C-terminal domain. The intradermal response (IDR) against *L. donovani* antigen and the increase of TNF-α, when compared to IL-10, expressed by CD4^+^ lymphocytes were very good correlates of vaccine induced immunity (48). Important epitopes for mice (48), human, and dog B cells (49) were also recently demonstrated in the sequence of the C-terminal domain.

In the search for cross-protection for CL leishmaniasis, we further vaccinated mice with the NH36 domains and challenged them with *L. amazonensis* (48). Different from the absolute dominance of the C-terminal domain in immune protection to VL, the most severe syndrome (1), preliminary results suggest that protection against CL by *L. amazonensis* is mediated by the C-terminal and the N-terminal domain in similar proportions (48).

In the present work, we studied the immunotherapeutic effect of NH36 or its peptide components in a formulation with saponin, on mice infection by *L. amazonensis*, in order to assess which of the NH36 domains deserves to be considered as components in a future cross-therapeutic vaccine for leishmaniasis. We identified that the N-terminal and C-terminal domains of NH36 induced strong curative effects which improved, as the CD4 T cell responses shifted from single- to double-cytokine producers (TNF-α^+^-IL-2^+^), and, in the case of the N-terminal domain vaccine, as the CD8 T cell responses shifted qualitatively from single- to triple-cytokine producers (TNF-α^+^-IL-2^+^-IFN-γ^+^).

## Materials and Methods

### Ethical statements

All experiments were reviewed and approved by the Animal Care and Use Committee of the Instituto de Biofisica Carlos Chagas Fo.-UFRJ (CAUAP-CONCEA, Brazil, IMPPG-016) and were performed according to the guidelines of the National Institutes of Health, USA. We made all efforts to minimize animal suffering.

### Nucleoside hydrolase-NH36 domains

The sequence of DNA and amino acids of NH36 is deposited in the EMBL, GenBank™, and DDJB data bases, access number AY007193. NH36 is composed of 314 amino acids. The three peptide domains of NH36 codifying, respectively, for the amino acids 1–103 (F1), 104–198 (F2), and 199–314 (F3) were cloned in the pET28b plasmid and were expressed and chromatographed as previously described (48). A preliminary molecular model was obtained through homology modeling using the Modeller9.10 software and the data of the nucleoside hydrolase from *L. major* template (RCSB PDB code: 1EZR; Crystal structure of nucleoside hydrolase of *L. major*) (50). It is important to note that the model shown in this investigation is preliminary and not a final, optimized model. The predicted epitopes for MHC class II-IAd and IEd, haplotype H2d CD4^+^ T cells, MHC class I Ld-CD8^+^ T cells, and B cells were plotted in the C-terminal and N-terminal moieties of the model (48). Additionally, the analysis of the solvent accessible surface area of the C-terminal, central, and N-terminal sections of the tetramer was performed using the PyMol 1.3 software.

### Immunotherapeutic vaccination in *Leishmania amazonensis* infected mice

Two-month-old Balb/c mice (female) were infected with 10^5^ *L. amazonensis* (pH 8 strain) metacyclic promastigotes isolated from hamsters and maintained in Schneider’s medium in the right hind footpads (45). The evolution of lesions was monitored weekly with a caliper apparatus (Mitutoyo) and the swelling of the non-infected contra-lateral left footpads were subtracted. Six weeks after infection groups of mice received three doses of 100 μg of NH36, F1, F2, or F3 recombinant proteins and 100 μg of SIGMA saponin (NH36sap, F1sap, F2sap, and F3sap vaccines, respectively), at weekly intervals, in the back by the sc route, while the control group was treated with saline solution. At 9 weeks after infection sera were collected for the assay of anti-NH36 antibodies in an ELISA assay and the IDR against *L. amazonensis* (pH 8) lysate (IDR) was determined in the footpads as described previously (48). Mice were euthanized with CO_2_ and their cellular immune response was assessed by intracellular staining (ICS), multiparameter cytometry analysis of splenocytes (51, 52), and by a cytokine-ELISA assay of the splenocytes supernatants. The total number of parasites in the footpad lesions was determined after sacrifice by Real Time PCR as previously described (53) using primers for *L. chagasi* on DNA isolated from infective promastigotes of *L. amazonensis* (pH 8) obtained from hamsters footpads (48).

### Assessment of the cellular immune response

The cellular immune response was assessed using 10^6^ splenocytes that had been cultured in RPMI for 72 h *in vitro* at 37°C and 5% CO_2_ in the presence or absence of 5 μg of NH36. The multiparameter analysis (51, 52) was carried out to assess the intracellular production of IL-2, TNF-α, and IFN-γ cytokines by CD4^+^ and CD8^+^ T lymphocytes. For this evaluation, the cells were treated with brefeldin (SIGMA) at a final concentration of 10 μg/ml, incubated for an additional 4 h, and then stained with rat anti-mouse CD4FITC (clone GK1.5) and CD8FITC (clone 53–6.7) monoclonal antibodies (R&D systems, Inc.) and further stained with IFN-γAPC, IL-2-PerCP-Cy5.5, and TNF-αPE monoclonal antibodies (BD-Pharmingen) as described before (48). For the ICS methods, 100,000 lymphocytes were acquired using a BD FACScalibur apparatus. Data were analyzed using the Cell Quest program. The secretion of cytokines was also evaluated in the supernatants of splenocytes by an ELISA assay as previously described (48).

### Statistical analysis

The Kruskal Wallis and Mann Whitney non-parametrical tests were used for comparison of means and the two-tailed Pearson bivariate analysis for the assessment of the correlation coefficient (GraphPad Prism 6 for Windows).

## Results

**Figure 1:**
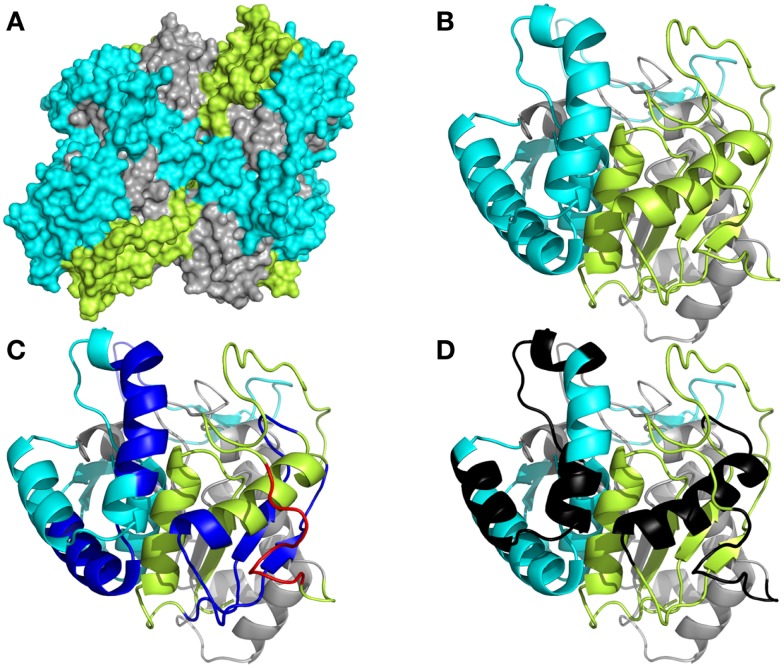
**Spatial distribution of epitopes in the monomer of *Leishmania donovani* nucleoside hydrolase-NH36**. **(A)** Illustration of the tridimensional surface model of the NH36 tetramer obtained by homology modeling to the sequence of the nucleoside hydrolase of *L. major*. **(B)** Monomer of *L. donovani* NH36 with the sequences of the N-terminal (F1, amino acids 1–103 in lime green), central (F2, amino acids 104–198 in gray), and C-terminal (F3, amino acids 199–314 in cyan) moieties. **(C)** MHC class II-IAd and IEd, haplotype H2d CD4^+^ T cell epitopes (dark blue), and of MHC class I Ld-CD8^+^ T cell predicted epitopes (red) of the C-terminal and N-terminal moieties. **(D)** Epitopes for B cells on the C-terminal and N-terminal moieties (black).

To understand more clearly how the peptide domains F1, F2, and F3 are distributed along the whole of the NH36 molecule, we obtained the preliminary model of the nucleoside hydrolase-NH36 by homology to the model of the nucleoside hydrolase of *L. major* (Figure 1). Our aim was only to illustrate the molecule. The image of the molecule shows its tetramer composition, with four identical subunits. The solvent accessible surface area was computed and this disclosed the distribution of the F1, F2, and F3 domains (Figure 1A). This tridimensional surface model of the tetramer revealed that the F3 (C-terminal domain) is the domain with the largest area of exposed surface (29,507,002 Å) (Figure 1A). This is followed by the F1 (N-terminal domain) with an area of 27,132,781 Å. The F2 (central domain) has the smallest surface area (19,931,451 Å) and is therefore the least exposed domain (Figure 1A). The detailed monomer (Figure 4B) shows the F3 as the most exposed peptide, followed by the less exposed F1, while the F2 fragment (central domain), on the other hand, is apparently more hidden (Figure 4B). The sequence of F3 includes three predicted epitopes for CD4^+^ T cells (Figure 4C) and three epitopes for antibodies (Figure 4D) while the F1 shows two epitopes for CD4^+^, one epitope for CD8^+^ T cells (Figure 4C), and two epitopes for antibodies (Figure 4D).

**Figure 2:**
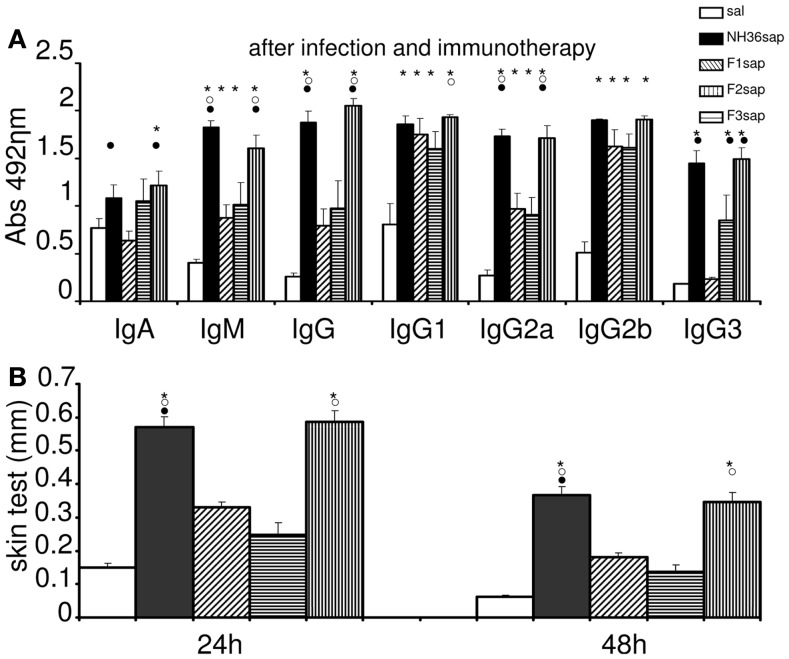
**Therapeutic vaccination, anti-NH36 antibodies, and intradermal response to *L. amazonenis***. Six weeks after infection with 10^5^ metacyclic promastigotes of *L. amazonensis* in the footpads, Balb/c mice were further vaccinated with three subcutaneous doses of NH36sap, F1sap, F2sap, or F3sap at weekly intervals. Bars represent the mean ± SE of the absorbance values of anti-NH36 antibodies from 1/100 diluted serum **(A)** and intradermal response to the promastigote lysate of *L. amazonensis* (24 and 48 h after antigen injection) **(B)** of two independent experiments with *n* = 6–7 mice per treatment performed after complete vaccination. *p < 0.05 different from the saline control; ^∘^*p* < 0.05 different from the F2sap vaccine; ^•^from the F1sap vaccine.

We also studied the immunotherapeutic effect of the NH36, F1, F2, and F3-saponin vaccines in mice previously infected with *L. amazonensis*. On week 6 after infection, when significant differences between the sizes of infected and the contra-lateral uninfected footpads were already detected, three doses of each vaccine were injected with weekly intervals. Sera samples were obtained and analyzed for anti-NH36 antibodies 1 week 9 after completing vaccination schedule (Figure 2A). Significant variations were detected for all antibody classes and subtypes (*p* < 0.0001). The F3sap vaccine induced levels of anti-NH36 IgA, IgM, IgG, and IgG2a antibodies as high as the NH36 vaccine and of IgG1 antibodies higher than the F2sap vaccine indicating that the main NH36 B cell epitopes involved in immunotherapy are located in the C-terminal moiety of NH36. The F1 vaccine, on the other hand, induced only IgG2b levels higher than saline controls and compatible with all other vaccines (Figure 2A).

After immunotherapy, the IDR specific response against *L. amazonensis* lysate was predominant in the F3 vaccinated mice, which showed an IDR as high as the one induced by the NH36 vaccine (Figure 2B). The other peptide vaccines were not different from the saline treated controls which exhibited, as expected for CL leishmaniasis, a positive and mild IDR reaction of 0.15 mm at 24 h and 0.06 mm at 48 h (Figure 2B). This result points out the pre-dominance of the epitopes present at the C-terminal domain in the generation of a cellular immune response to *L. amazonensis* infection.

**Figure 3:**
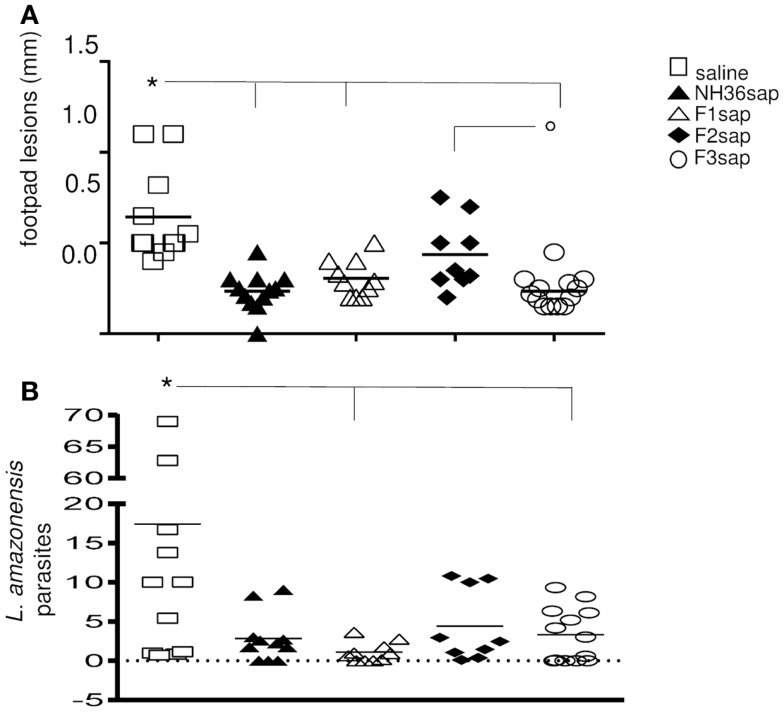
**Therapeutic effect of NH36 vaccines on *L. amazonensis* infection**. **(A)** Individual sizes of footpad lesions 9 weeks after infection with 10^5^ infective promastigotes of *L. amazonensis* and further treated with NH36, F1, F2, and F3 vaccines formulated in saponin. Results represent the mean + SE of the footpad measurements of two independent experiments (six to seven animals per treatment in each experiment). *p < 0.05 different from the saline control and ^∘^*p* < 0.05 different from the F2sap vaccine. **(B)** *Leishmania amazonensis* promastigotes in the footpad lesions as determined by Real Time PCR. *Lines indicate significant differences compared to the saline control (*p* < 0.05).

As a further measure of the therapeutic effect, we compared lesion development and parasite burden on week 9 after challenge. Significant differences between treatments were detected in the size of the footpad lesions along the time (*p* < 0.0001). The NH36sap (*p* < 0.001), F1sap (*p* < 0.05), and F3sap vaccines (*p* < 0.001) reduced the size of footpad lesions, along the time, to a similar extent if compared to the untreated infected saline controls. The F3sap vaccine also showed to be more therapeutic than the F2sap vaccine (*p* < 0.05) (not shown). When looking in detail at the individual footpad sizes on week 9 (Figure 3A), it was possible to observe that the best therapeutic effect was detected in the F3sap vaccinated mice, whose mean lesion size (0.23 mm) was 64% (*p* < 0.001) lower than that of the saline controls (0.64 mm) and 48% (*p* < 0.05) lower than that of the F2sap vaccine group (0.44 mm) (Figure 3A). The sizes of footpad lesions at week 9 were significantly correlated to the number of *L. amazonensis* parasites in lesions quantified by RTPCR, which disclosed that only the N-terminal and C-terminal domains reduced to 82.6% (*p* < 0.006) and 81% (*p* < 0.021), respectively, the number of parasites in lesions when compared to the control animals (Figure 3B). No difference in parasite load was detected between both vaccines (*p* < 0.05). Mice treated with F2sap, on the other hand, showed no decrease in parasite load when compared to the untreated controls (*p* > 0.05) (Figures 3A,B). None of the animals in the saline control or F2 vaccine group showed a total absence of parasites, however, three animals of the F1 and NH36 vaccines, and two animals of the F3 vaccine showed zero parasites in their footpad lesions.

**Figure 4:**
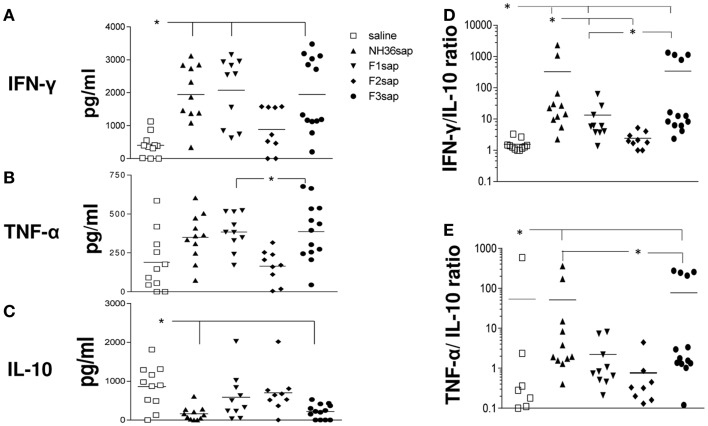
**Cytokine expression**. After euthanasia, the secretion of IFN-γ, TNF-α, and IL-10 were evaluated by an ELISA assay, in the supernatants of splenocytes, which had been incubated with NH36 for 72 h. Results in **(A–C)** are presented as means and individual levels of secreted cytokines, expressed as picogram per milliliter, of two independent experiments (six to seven mice per treatment in each experiment) and as the IFN-γ/IL-10 **(D)** and TNF-α/IL-10 **(E)** ratios. *Significant differences between treatments.

Furthermore, the cytokine levels secreted to the splenocytes supernatants after stimulation with NH36 were measured (Figure 4) and significant variations among treatments were detected for the secretion of IFN-γ (*p* < 0.001), TNF-α (*p* < 0.001), and IL-10 (*p* < 0.01) (Figures 4A–C). The NH36sap, F1sap, and F3sap vaccines induced increased levels of IFN-γ above the saline controls (*p* < 0.01 for each vaccine) (Figure 4A) while TNF-α was increased by the F3sap (*p* < 0.05) and F1sap (*p* < 0.05) above the F2sap vaccine (Figure 4B). Additionally, only the NH36sap (*p* < 0.01) and F3sap (*p* < 0.05) vaccines expressed lower levels of IL-10 than the saline controls (Figure 4C). Therefore, while the F3 vaccine promoted a TH1 therapeutic response with high secretion of the pro-inflammatory cytokines IFN-γ and TNF-α and low levels of the regulatory cytokine IL-10, the F1 vaccine, differently, induced the increase of IFN-γ and TNF-α (Figures 4A,B) but no decrease however, of the IL-10 levels (Figure 4C). The analysis of the IFN-γ/IL-10 ratio (Figure 4D) disclosed also that the F2sap vaccine did not differ from the saline control. The higher ratios were seen in animals treated with the NH36 and F3 vaccines, followed by the F1sap vaccine (Figure 4D). The TNF-α/IL-10 ratios, on the other hand were only enhanced by the NH36sap and F3sap vaccine above the levels of the saline controls and F2sap vaccine (Figure 4E).

**Figure 5:**
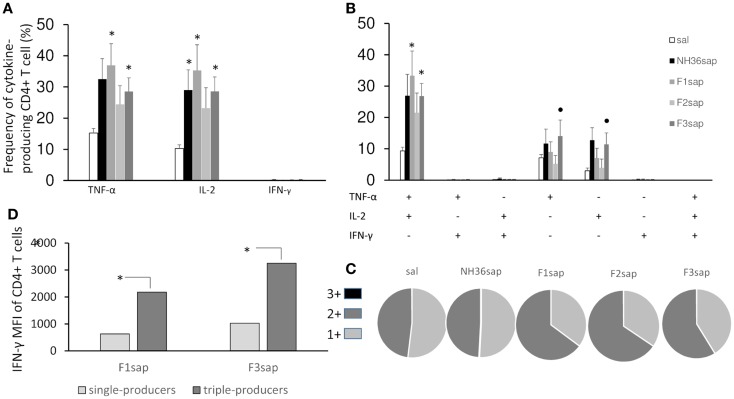
**Multifunctional analysis discloses the magnitude and quality of the CD4^+^ T cell response**. NH36-specific cytokine production from CD4^+^ T cells of spleens of immunotherapy treated and control mice 9 weeks after infection **(A–C)**. Multiparameter flow cytometry was used to determine **(A)** the total frequency of IFN-γ-, IL-2-, or TNF-α-producing CD4^+^ T cells, **(B)** the frequency of cells expressing each of the seven possible combinations of IFN-γ, IL-2, and TNF-α, **(C)** the magnitude of the IFN-γ secretion expressed by its median intensity fluorescence (MFI) in single- and triple-cytokine CD4^+^ T cell producers and **(D)** the fraction of the total response comprising cells expressing all three cytokines (3+), any two cytokines (2+), or any one cytokine (1+). Results shown as the mean ± SE of two independent experiments with *n* = 6–7 in each experiment. *Significant differences from saline treated controls, •significant differences from the F2sap vaccine.

Based on the requirements of IFN-γ and the roles of TNF-α and IL-2 as effector cytokines that mediate protection, we assessed the frequency of NH36-specific IFN-γ, IL-2-, and TNF-α-producing CD4^+^ T cells after immunotherapy treatment by multiparameter cytometry analysis. We initially assessed the total frequencies of CD4^+^-T cells producing IFN-γ, IL-2-, and TNF-α, which summarize the frequency of cells that produce each particular cytokine alone (single producers), and together with one more (double producers) and two other cytokines (triple cytokines). On week 9 after infection, significant differences between treatments in the total frequencies of TNF-α (*p* = 0.0077) and IL-2-producing-CD4^+^ T cells of the spleens (*p* = 0.0035) were found (Figure 5A). The total frequencies of CD4^+^ T cells producing TNF-α and IL-2 (Figure 5A) were significantly increased above their saline controls and reached 35–36%, in the case of F1sap, and 28% in the case of the F3sap vaccine, while the NH36 vaccine increased only the IL-2-producing cells to 29%. In agreement with that, the proportion of CD4^+^ T cells producing TNF-α^+^-IL-2^+^ was increased to 33 and 27%, by the F1sap and F3sap vaccines, respectively (Figure 5B). On the other hand, the frequencies of IL-2^+^ and TNF-α single cytokine producer CD4^+^ T cells were increased significantly, only by the F3sap vaccine to 14 and 12%, respectively (Figure 5B).

In contrast to the lack of correlation seen by measuring the total frequencies of CD4^+^IFN-γ^+^ producing cells alone or in combination with other cytokines (Figures 5A,B), which collectively developed frequencies below 1%, our analysis showed a high correlation between the frequency of multifunctional (IL-2, TNF-α, TNF-α-IL-2) CD4^+^ T cells and the degree of protection. The sizes of footpad lesions (Figure 3A), which were positively correlated with the number of parasites (Figure 3B) (*R* = 0.7239, *p* < 0.001), were negatively correlated to the total frequencies of CD4^+^-IL-2^+^ (*R* = −0.3063; *p* = 0.0243), CD4^+^-TNF-α^+^ (*R* = −0.2847; *p* = 0.0369), CD4^+^-IL-2^+^-TNF-α^+^ (*R* = −0.2964; *p* = 0.0295) and of the CD4^+^-IL-2^+^ (*R* = −0.3611; *p* = 0.0068) single cytokine producer T cell populations (Figures 5A,B).

Differences in the quality of the response between vaccine groups are represented pictorially by pie charts (Figure 5C). Quantifying the fraction of the total cytokine response comprising three (3+), any two (2+), or any one (1+) cytokine, we found that over a half of the CD4^+^-responses in untreated controls, NH36sap and F3sap vaccines were 1+ cells, while 65% of the response in F1sap and F2sap vaccines were 2+ cells.

Remarkably, and despite the low global frequency of triple-cytokine and of IFN-γ producing CD4^+^ T cells (Figures 5A,B), we noted a progressive 3,165 and 3,473-fold increase in the median fluorescence intensity (MFI) for IFN-γ from CD4^+^ T cells that secrete all the three cytokines compared with single cytokine-producing CD4^+^ T cells (Figure 5D) only in the animals treated with the F1sap and the F3 vaccines.

**Figure 6:**
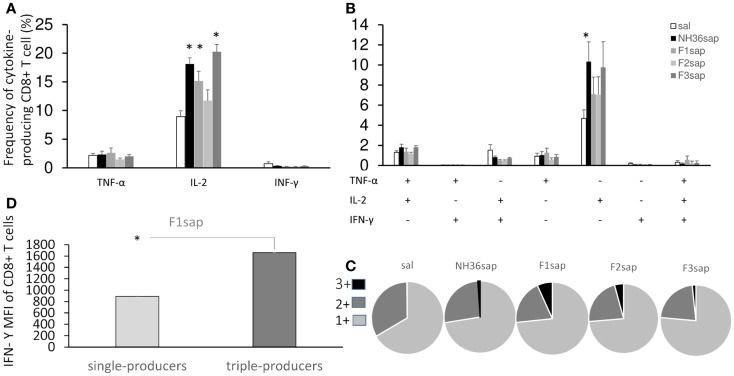
**Multifunctional analysis discloses the magnitude and quality of the CD8^+^ T cell response**. NH36-specific cytokine production from CD8^+^ T cells of spleens of immunotherapy treated and control mice 9 weeks after infection **(A–C)**. Multiparameter flow cytometry was used to determine **(A)** the total frequency of IFN-γ-, IL-2-, or TNF-α-producing CD8^+^ T cells, **(B)** the frequency of cells expressing each of the seven possible combinations of IFN-γ, IL-2, and TNF-α, **(C)** the magnitude of the IFN-γ secretion expressed by its median intensity fluorescence (MFI) in single cytokine and triple-cytokine CD8^+^ T cell producers and **(D)** the fraction of the total response comprising cells expressing all three cytokines (3+), any two cytokines (2+), or any one cytokine (1+). Results shown as the mean ± SE of two independent experiments with *n* = 6–7 in each experiment. *Significant differences from saline treated controls.

On the other hand, the multiparameter analysis of the NH36-specific CD8^+^ T cell population, disclosed that the total frequencies of IL-2-producing cells were enhanced to 19, 15, and 20%, respectively, by the NH36, the F1sap, and the F3sap vaccines (Figure 6A). The frequency of IL-2^+^ single cytokine producer CD8^+^ T cells was increased (Figure 6B) above controls and to 10%, by the NH36 vaccine. In correlation with that, the multifunctional analysis revealed that only the increase of the CD8^+^ T cells producing IL-2 or TNF-α or both were predictive of the therapeutic effect of vaccination. Indeed, the total frequencies of CD8^+^-IL-2^+^ (*R* = −0.4575; *p* = 0.004 and *R* = −0.4363; *p* = 0.0292), CD8^+^-IL-2^+^-TNF-α^+^ (*R* = −0.2795; *p* = 0.0407 and *R* = −0.3820; *p* = 0.0500) and the CD8^+^-IL-2^+^ single cytokine populations (*R* = −0.3716; *p* = 0.0057 and *R* = −0.5367; *p* = 0.0057) were negatively correlated to the sizes of footpad lesions and the number of parasites in lesions, respectively.

Furthermore, quantifying the fraction of the total cytokine response of CD8 T cells comprising three (3+), any two (2+), or any one (1+) cytokine (Figure 6C), we found that while almost no triple labeled cells were detected in the saline controls (mean = 0.31%), this proportion increased in all vaccinated groups and exhibited the highest values in the F1sap vaccinated mice (6.75%). We also found that 72–76% of the response to all treatments was 1+ cell and from 20 to 32% was 2+ cell. The proportion of the 3+ labeled CD8+ T cells increased therefore at the expense of the 2+ cell population (Figure 6C).

Regarding the magnitude of the immune response and in agreement with the highest frequency of triple-producers cells in F1sap vaccinated mice (Figure 6C), we noted a 1,859-fold increase in MFI for IFN-γ in CD8^+^ T cells that secrete all the three cytokines when compared to the single cytokine-producing CD8^+^ T cells (Figure 6D), only in mice treated with the F1sap vaccine (Figure 6D).

**Figure 7:**
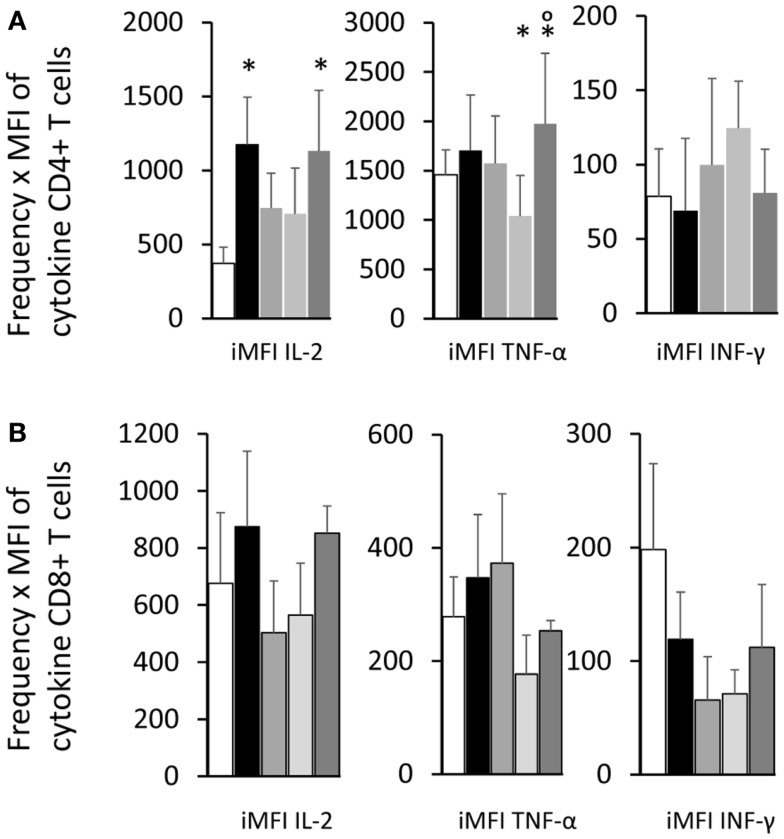
**Total functional response of the single cytokine producer T cells**. By multiplying the frequency by the MFI of the single cytokine producer T cells, we calculated the iMFI that reflects the total functional response of the population. Single cytokine producer CD4^+^ **(A)** and CD8^+^ T cells **(B)**. Results shown as the mean ± SE of two independent experiments with *n* = 6–7 in each experiment. *Significant differences from saline treated controls.

As an alternative method to calculate the magnitude of the response, the iMFI values were additionally obtained by multiplying the frequency of the single cytokine producer CD4^+^ T cells and their MFI of single cytokine producers (Figure 7A). The F3 vaccine enhanced the iMFI-IL-2 and, together with the NH36 vaccine, also the iMFI-TNF-α values over the respective saline controls. There was not any significant variation in the magnitude of the response (iMFI) of CD8^+^ T cells for any cytokine by any of the vaccines (Figure 7B).

Collectively, our multifunctional analysis revealed that the immunotherapy treatment with NH36 peptide vaccines determined that the IL-2, TNF-α, and TNF-α-IL-2-CD4^+^ and -CD8^+^ T cells were predictive of protection and immunotherapeutic potential of the vaccines and that protection improved as the CD4 responses shifted from 1+ to 2+ and the CD8 responses shifted qualitatively from 1+ to 3+.

## Discussion

Several epitopes for T cell lymphocytes and antibodies where predicted along the whole sequence of NH36 but they have different levels of immunogenicity in prophylaxis against *L. chagasi* infection (48). The calculation of the surface area of the NH36 model revealed that the sequences of the F3 and F1 peptides are the most exposed and this suggests they have a greater availability for lysosome or proteaimmunosome enzymes, and hence, the enhanced probability of being presented by the MHC receptors. The F3 peptide, which has the highest number of predicted epitopes for CD4^+^ T cells and antibodies (48), has the largest surface area and, is the target of the strongest cellular and humoral immune response against *L. amazonensis* (in this investigation) and *L. chagasi* (48). On the other hand, the lower access of the F2 domain to the surface area explains its lower immunogenicity in the *L. chagasi* (48) and *L. amazonensis* infection models, despite the prediction of one epitope for CD4^+^, two for CD8^+^ T cells, and two for antibodies in its sequence (48).

After immunotherapy of *L. amazonensis* infection, only the F3 vaccine stands out as the most potent inducer of IgG and IgG2a anti-NH36 antibodies, while the IgG2b and IgG1 antibodies were equally enhanced by the F1, F2, and F3 vaccines. Interestingly, the F1 vaccine was less capable than the F3 vaccine in sustaining the IgG2a response. Additionally, the F2 vaccine induced an increase in IgG1 and IgG2b antibodies, which indicates the advancement of infection and is not correlated to therapeutic protection. We conclude that the most important epitopes for anti-NH36 antibodies generated after immunotherapy of *L. amazonensis* or prophylaxis against *L. chagasi* infection (48) are located in F3. F3 is then the target of the anti-*Leishmania* cross-specific humoral response of mice (48), and the antibody target of humans and dogs with VL (49) and of dogs vaccinated with Leishmune^®^ (17). Since the antibodies generated by the Leishmune^®^ vaccine in dogs reacted mostly with the F3 epitopes (48) and block the transmission of VL in the insect vector (18, 19), the identification of these cross-reactive immunogenic sequences in F3 might also help in blocking the transmission of CL.

The IDR to the lysate of *L. amazonensis* after immunotherapy was enhanced by the F3 and NH36 vaccines, similarly to what was detected before and after infection by *L. chagasi* (48). IDR is a well known correlate of protection that is expected to be absent in patients with VL (6) and DCL (7), who show immunosuppression, but present in cured individuals (6, 7), or after generation of vaccine protection (16, 17, 48, 54, 55) or in patients with CL caused by *L. braziliensis*, which, on the contrary, show a strong TH1 response (8). In the selection of candidates for clinical trials of vaccines against CL, IDR is the main criteria for exclusion, as it indicates sensitization due to previous contact with the parasite (56). The description of the F3 vaccine and the NH36 vaccine as good enhancers of the IDR response of mice infected with *L. amazonensis* infection is important for the future development of defined cross-protective vaccines since: (1) *L. amazonensis* causes both CL and DCL (2, 7) individuals with DCL are commonly anergic, showing diminished or absent immune responses to *Leishmania* antigens (3, 7) and (3) the single human vaccine licensed for immunotherapy of CL leishmaniasis is based on a *L. amazonensis* crude vaccine (15).

Additionally, as described for mice prophylaxis against VL and CL (48) the F3 vaccine was the most therapeutic against *L. amazonensis*, reducing the size of footpad lesions and the parasite load. A significant, although different, therapeutic effect was induced by the F1 vaccine. While both vaccines induced high secretion of the pro-inflammatory cytokines IFN-γ and TNF-α by splenocytes, only the F3 vaccine exhibited the typical TH1 response with reduced levels of IL-10. The epitope prediction programs disclosed the existence of three epitopes for CD4^+^ in the F3 and two in the F1 sequences, respectively. The CD8^+^ T cell epitope prediction program disclosed the highest affinity for the YPPEFKTKL epitope in F1 and no epitope in F3 (48). Accordingly and as described for VL (48), an *in vivo* depletion assay recently demonstrated that, protection against *L. amazonensis* infection is mediated by a TH1 CD4^+^ T cell driven response to F3 and a CD8^+^ T cell mediated response to the F1 domain (57).

In agreement with the above mentioned responses, Seder et al. (51), when describing immune correlates for vaccine-elicited protection against CL, stated that a CD4^+^ T-Helper 1-type response is considered necessary and even sufficient for infection by *L. major*, while CD8^+^ T cells are considered to have an important role in protection following natural infection and may be important for optimizing vaccine efficacy. Their model involves the earliest single secretion of TNF-α and of IL-2, followed by the development of double producers (TNF-α and IL-2) and by the later triple-producers of IFN-γ-TNF-α-IL-2 that can persist as memory or effector CD4^+^ T cells. In agreement with that our work revealed as a correlate of protection, the increase of total frequencies of TNF-α and IL-2, single and double producers of IL-2-TNF CD4^+^ T cells while the work of Darrah et al. (52) indicated the triple-positive CD4^+^ T cells. This fact could suggest that the MML of *L. major* live vaccines promotes a more mature condition of immune protection. However, while Darrah et al. (52) described the correlation between the triple-cytokine producers and protection only post-vaccination, our analysis disclosed the correlates after challenge with *L. amazonensis* and immunotherapy. Indeed, there is no description of immune correlates for the protection by the MML vaccine after challenge with *L. major* (52). It is worth noting that it is more difficult to generate protection and disclose the immune correlates after the establishment of infection than before. Furthermore, while Darrah et al. (52), used cells of draining lymph nodes of C57BL/6 mice, inoculated with *L. major* intradermally in the ear, we used splenocytes of Balb/c mice inoculated with *L. amazonensis* in the footpads. Our approach reveals the state of the systemic cellular immunity. The different antigen and adjuvant composition of the vaccines could also account for the differences.

In contrast to Darrah et al. (52), we did not find a correlation between the frequencies of IFN-γ^+^-producer cells and protection, and their frequencies were very low. In agreement with Darrah et al. (52), however, after immunotherapy with the F3 and F1 vaccines, we observed a progressive increase in the MFI values of IFN-γ as the degree of functionality increased from single- to triple-cytokine producers, indicating that IFN-γ might also represent a contribution to the cellular immune response against *L. amazonensis*. Another reason for the detection of low frequencies of IFN-γ-CD4^+^ producers by ICS might be the time of *in vitro* incubation. After 72 h, the IFN-γ might have already been secreted and therefore would no longer be inside the cells. The detection of increased amount of IFN-γ in the supernatants of the same cells, of mice treated with the NH36, F1, or F3 vaccines confirms that hypothesis. Furthermore, the time of *in vitro* incubation might also be the reason for the higher frequencies of CD4^+^ (30–40%) and CD8^+^ cytokine producers cells (20%). In the work of Darrah et al. (52), *in vitro* incubation lasted for 2 h only and the frequencies of T cells ranged from 0 to 1%.

An important role of the CD8^+^ T cells in protection against CL (51, 58) and VL (59) has been reported. Although the NH36 vaccine induced a CD4^+^ T cell mediated protection or therapy against VL in mice (43, 47) and dogs (46, 60), the recombinant NH36-saponin vaccine showed equal contributions of CD4^+^ and CD8^+^ T cells in protection for mice against VL (48) with the F3 being responsible for the CD4^+^ response to VL (48) and CL and the F1 being responsible for the main CD8^+^ T cell driven protection against *L. amazonensis* (57).

According to Seder et al. (51), following activation, the naïve CD8^+^ T cells fully differentiate into activated effector CD8^+^ T cells that secrete IFN-γ, most with cytolytic activity, which can further differentiate into CD8^+^ T effect memory cells (T_EM_) secreting IFN-γ–TNF-α, either directly, or after a step of conversion, to CD8^+^ central memory cells (T_CM_) which are triple–cytokine producers (IFN-γ^+^-TNF-α^+^-IL-2^+^). Therefore, the induction of IL-2 in CD8^+^ T cells is detected at a later time and is lost in chronic infections (51). In contrast to CD4^+^ T cells, it is considered very rare to find CD8^+^ T_EM_ cells that produce IL-2. The enhanced ability of CD8^+^ T_CM_ cells to produce IL-2 has been shown to confer improved protection compared with CD8^+^ T_EM_ cells against a systemic viral challenge (61). Our results of immunotherapy of *L. amazonensis* infection with the F1-saponin vaccine gain relevance since frequencies of total and single IL-2^+^ CD8^+^ T cell producers were significantly increased, were predictive of the therapeutic effect and the percentages of triple-cytokine producers were also increased. We recently demonstrated that protection against *L. amazonensis* infection is mediated by the CD8^+^ T cell response induced by the F1 vaccine (57). Williams et al. (62) showed the IL-2 signaling to pathogen-specific CD8^+^ T cells is required for the generation of robust secondary responses, programing the development of CD8^+^ memory T cells capable of full secondary expansion. Our results suggest that the F1 domain, which contains the highest affinity epitope of the NH36 for CD8^+^ T cells (48), might be important for the development of CD8^+^ T_CM_ cells (51), which through the high secretion of IL-2, or TNF or IL-2-TNF actively contribute to the cure of the established infection. The intensity of IFN-γ secretion by triple-producers, in our investigation, also proved to be above the levels of single producers, indicating the progressive increase in the MFI values of IFN-γ in F1sap treated mice as the degree of functionality increased.

The F1sap vaccine was also a determinant in the increased secretion of IFN-γ by CD4^+^ triple-cytokine producers, in the increased frequencies of total and double TNF-α and IL-2 producers and in the secretion of IFN-γ and TNF-α into the splenocyte supernatants indicating the induction of a TH1 response. However, mice treated with the F1 vaccine also showed a high secretion of IL-10 by splenocytes. While in VL IL-10 is considered to be the marker of the severe immunosuppressive disease (5, 63), IL-10 in human CL has been shown to be related to the pathology of the disease as well as the control of the parasite (64). Recently, the frequency and functional capacity of Tregs were evaluated in chronical patients with CL and in asymptomatic subjects (65). Although, the chronical patients presented higher frequencies of Tregs in peripheral blood and higher expression of *FOXP3* at leishmanin skin test sites, their CD4^+^CD25^+^ cells were less capable of suppressing antigen specific IFN-γ secretion by effector cells compared with asymptomatic infected individuals. At the end of the treatment, both the frequency of CD4^+^CD25^hi^CD127^−^cells and their capacity to inhibit proliferation and IFN-γ secretion increased and coincided with healing of CL lesions suggesting that the restored IL-10 secretion by Tregs was involved in the cure of the disease (65). The authors suggested that the Tregs impaired function was evidence of pathogenesis of CL and Treg subsets would be relevant in designing immunotherapeutic strategies for recalcitrant dermal leishmaniasis (65). CD4^+^CD25^+^ regulatory T cells have also been shown to restrain pathogenic responses during *L. amazonensis* infection (66). The simultaneous induction of an immunotherapeutic effect and the increase in the secretion of IL-10 determined by the F1 peptide might also be related to the stimulation of Treg subsets and to the presence of epitopes for Tregs along its sequence.

In our investigation, a significant decrease of IL-10 levels was found in the supernatants of whole splenocytes of F3 vaccinated mice. A population of IFNy^+^-producing CD4^+^ T cells that also produce IL-10 has been identified in VL as a feature of T cell differentiation (67). Expanded numbers of these cells were associated with disease progression (67). Conventional CD11c^hi^ DCs that produce both IL-10 and IL-27 It have also been shown to promote the production of IL-10 by these effector CD4^+^ T cells (67). In our investigation, besides CD4^+^ T cells, DCs could also be the source of the IL-10 secretion detected in splenocyte supernatants. These types of DCs were also present in our mice model of CL infection. If that is the case, we could assume that immunotherapy with the F3 peptide formulated with saponin, could promote the direct shifting of DCs away from an IL-10 producing phenotype, which is more frequent in the untreated controls, to a pro-Th1 IL-12 producing phenotype, with reduced IL-10 secretion (67).

An alternative source of IL-10, in *L. amazonensis* infected mice, could be natural killer (NK) cells. In mice infected with *L. donovani*, NK cells are found in the spleen and liver hepatic granulomas (68). They are responsible for suppressing the host resistance to the parasite, through the secretion of IL-10, which is present in early infection. In mice with an established infection, the IL-10 mRNA acquires more stability and IL-10 secretion by NK is enhanced (68). In the context of CL leishmaniasis, IL-10 has been shown to be essential for *L. major* persistence (69). NK cells were also more frequent in relapsed than in cured cases of mucosal leishmaniasis and a decrease in NK cells and in IL-10 levels was observed after therapy (70).

A few other antigens have been proposed as potential synthetic vaccines against leishmaniasis (59, 71–73). The kmp-11 protein of *L. donovani* has epitopes recognized by human CD8^+^ lymphocytes and by many different HLA receptors (59). The Leish110f fusion protein of *L. major*, on the other hand, induced mice protection mediated by CD4^+^ lymphocytes (72). Recently, an adenovirus based vaccine comprising a synthetic HASPB gene composed of 10 repeats, linked to the KMP-11 gene, was obtained and assayed in the therapy of *L. donovani* infected mice therapeutics (73). The synthetic gene was cloned using humanized codons. The immunogenicity increased if the vaccine was administered in the footpads instead of subcutaneously. A detailed study of the contribution of the epitopes of HASPB protein was performed. After therapeutic vaccination, the IgG1 and IgG2a antibody responses were enhanced and IFN-γ-CD8^+^ T cell response, mainly to HASPB, became apparent. Interestingly, a single dose of the vaccine reduced the parasite growth in spleens by 66% (73).

Immunotherapy for the treatment of human VL leishmaniasis has recently been recommended (74). The C-terminal and N-terminal domains of NH36 of *L. donovani* are the basis of the strong immunotherapeutic effect against *L. amazonenis* infection. Our findings contribute to the design of defined vaccines for cross-protection against CL leishmaniasis.

## Author Contributions

Conceived and designed the experiments: Clarisa Beatriz Palatnik-de-Sousa, Dirlei Nico. Acquisition, analysis, and interpretation of data: Dirlei Nico, Daniele Crespo Gomes, Iam Palatnik-de-Sousa, Alexandre Morrot, Marcos Palatnik, Clarisa Beatriz Palatnik-de-Sousa. Wrote the paper: Clarisa Beatriz Palatnik-de-Sousa. Final approval of the last version of the manuscript to be published: Clarisa Beatriz Palatnik-de-Sousa, Dirlei Nico, Daniele Crespo Gomes, Iam Palatnik-de-Sousa, Alexandre Morrot, Marcos Palatnik.

## Conflict of Interest Statement

The authors have declared that there are no competing interests. This work is related to the pendent Patent PI1015788-3, INPI, Brazil.
